# How nicotine withdrawal symptoms fight each other: interpeduncular GABA neuron activity dynamically controls negative affect vs. coping behavior

**DOI:** 10.1038/s41386-021-01185-1

**Published:** 2021-09-30

**Authors:** Alicia J. Avelar, Olivier George

**Affiliations:** Department of Psychiatry, University of California, San Diego, La Jolla, CA 92093 USA

**Keywords:** Addiction, Excitability

Tobacco use remains the leading cause of preventable disease, disability, and death in the United States, accounting for 1 in 5 deaths. Nicotine, the main psychoactive component of tobacco, is largely responsible for the addictive properties of tobacco. Although nicotine may cause feelings of euphoria or stress relief, the withdrawal state of the nicotine dependence cycle causes symptoms such as increased anxiety, irritability, stress, physical discomfort, and a profound craving for nicotine. Nicotine withdrawal symptoms often result in relapse where the person seeks and consumes nicotine even if they want to quit smoking. Identification of the neural mechanisms underlying the effects of nicotine withdrawal is an essential step in understanding tobacco use disorder and may lead to better treatments. Converging evidence suggests that manipulation of GABAergic transmission in a small midbrain region enriched in nicotinic receptors, the interpeduncular nucleus (IPN), can control the emotional, physical, and motivational aspects of nicotine withdrawal. However, a critical gap in the literature is lack of evidence that GABA transmission is dynamically regulated during nicotine withdrawal in vivo.

The report by Klenowski and Zhao-Shea et al. [1], addresses this issue by using fiber photometry combined with cell-specific viral-mediated GCaMP expression to measure calcium activity in IPN GABA neurons of male mice experiencing nicotine withdrawal. Precipitated withdrawal using the nicotinic acetylcholine receptor antagonist mecamylamine resulted in increased GCaMP activity in IPN GABA neurons during nicotine withdrawal. This result confirms and extends previous findings demonstrating an increase in c-Fos (an immediate-early gene marker of neuron activity) in IPN GABA neurons during nicotine withdrawal [2]. However, a significant advance in the current report compared to previous studies using c-Fos is that fiber photometry is performed in vivo. The recording of in vivo neuronal activity during nicotine withdrawal is innovative and exciting because the data can be time-locked with the behavior, and therefore answer a question that could not be answered otherwise: what is the neuronal mechanism underlying the negative emotional state of withdrawal compared to the coping behaviors aimed at reducing this negative emotional state?

**Fig. 1:**
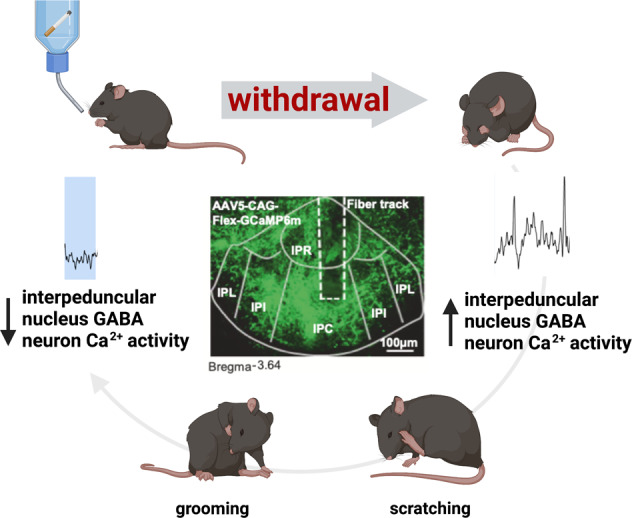
Relation between nicotine withdrawal, interpeduncular GABA neuron activity, and coping behavior. Withdrawal from nicotine produces withdrawal symptoms and increases interpeduncular nucleus GABA neuron calcium activity. Emergence of coping behaviors such as grooming and scratching reduces interpeduncular nucleus GABA neuron calcium activity.

For instance, based on the c-Fos studies, one could hypothesize that the increased IPN GABA neuron activity is due to an increase in the motivational and somatic signs of withdrawal. Indeed, the authors found that the activity of IPN GABA neurons was increased when the nicotine-dependent and withdrawn mice were exploring the open arms of an elevated plus-maze, a condition known to increase anxiety-like behavior. However, the detailed time-lock analysis by Klenowski and Zhao-Shea et al. [1], revealed that while nicotine withdrawal increases the overall activity of IPN GABA neurons, the expression of some of the withdrawal symptoms such as scratching and grooming behaviors was associated with a decrease in IPN GABA neuron activity. They also showed that optogenetic inhibition of IPN GABA neurons prevented the emergence of somatic signs of withdrawal, including scratching and grooming. These remarkable results demonstrate that the expression of grooming and scratching is not causing the increase in IPN GABA neuron activity per se, but instead that grooming and scratching behaviors decrease IPN GABA neuron activity. They also observed that the IPN GABA neurons activity tends to ramp up right before the grooming/scratching episode and to rebound right after the grooming/scratching episode ends, further suggesting that these withdrawal-related behaviors emerge as a coping mechanism to reduce the increase in IPN GABA neurons activity causing the anxiety-like behavior (Fig. 1).

Further investigation on the mechanisms and neural circuits underlying these coping behaviors may provide new targets for the development of medications that mimic the coping effect of these behaviors. It will be important in the future to identify which upstream circuits control these coping behaviors during withdrawal to decrease IPN GABA neuron activity. Two candidate upstream regions are the hypothalamus and the ventral tegmental area, which have been previously identified as key brain regions for the control of grooming behavior. However, very little work has been done in nicotine-dependent laboratory animals.

One limitation of the present report is that it was conducted in male mice only. The O’Dell lab has shown that female rats exhibit higher anxiety-like behavior and higher IPN GABA levels than males during nicotine withdrawaI [3], however, it is unknown if the increased IPN GABA neuron activity in female is also observed using calcium imaging. Moreover, it would be important to know if the increased IPN GABA neuron activity in females is due to a lower level of coping behavior, such as grooming and scratching, or if it leads to an even higher level of coping behaviors in females. Finally, since Carcoba et al., observed that this sex difference in IPN GABA levels was dependent on activation of corticotropin-releasing factor (CRF) 1 receptor in the IPN [3] and that a ventral tegmental area to IPN CRF pathway was previously identified by the Tapper group as a key modulator of nicotine withdrawal symptoms [4]. We speculate that activation of CRF1 receptor due to nicotine withdrawal-induced activation of CRF neurons in the ventral tegmental area [5] may contribute to the increased IPN GABA activity during nicotine withdrawal [1, 3].

Overall, the comprehensive study by Klenowski and Zhao-Shea et al. [1], provides compelling evidence for the role of IPN GABA neuron activity in modulating nicotine withdrawal and identify a complex and dynamic regulation of IPN GABA neuron activity during the expression of anxiety-like behaviors vs. coping behaviors during nicotine withdrawal.
